# Impact of Donepezil on Brain Glucose Metabolism Assessed Using [^18^F]2-Fluoro-2-deoxy-D-Glucose Positron Emission Tomography Imaging in a Mouse Model of Alzheimer’s Disease Induced by Intracerebroventricular Injection of Amyloid-Beta Peptide

**DOI:** 10.3389/fnins.2022.835577

**Published:** 2022-02-25

**Authors:** Gaëlle Hugon, Sébastien Goutal, Marie Sarazin, Michel Bottlaender, Fabien Caillé, Marine Droguerre, Mathieu Charvériat, Alexandra Winkeler, Nicolas Tournier

**Affiliations:** ^1^Université Paris-Saclay, Inserm, CNRS, CEA, Laboratoire d’Imagerie Biomédicale Multimodale (BioMaps), Service Hospitalier Frédéric Joliot, Orsay, France; ^2^Department of Neurology of Memory and Language, GHU Paris Psychiatry and Neurosciences, Paris, France; ^3^Faculté de Médicine, Université de Paris, Paris, France; ^4^NeuroSpin, Frédéric Joliot Life Sciences Institute, CEA, Université Paris-Saclay, Gif-sur-Yvette, France; ^5^Theranexus Company, Lyon, France

**Keywords:** PET imaging, Alzheimer’s disease, awake brain imaging, donepezil, Alzheimer’s mouse model, functional imaging (positron emission tomography)

## Abstract

Translational methods are needed to monitor the impact of the Alzheimer’s disease (AD) and therapies on brain function in animal models and patients. The formation of amyloid plaques was investigated using [^18^F]florbetapir autoradiography in a mouse model of AD consisting in unilateral intracerebroventricular (i.c.v) injection of amyloid peptide Aβ_25–35_. Then, an optimized positron emission tomography (PET) imaging protocol using [^18^F]2-fluoro-2-deoxy-D-glucose ([^18^F]FDG) was performed to estimate brain glucose metabolism: [^18^F]FDG was injected in awake animals to allow for 40 min brain uptake in freely moving mice. Anesthesia was then induced for 30 min PET acquisition to capture the slow and poorly reversible brain uptake of [^18^F]FDG. Impact of donepezil (0.25 mg/kg daily, 7 days, orally) on brain function was investigated in AD mice (*n* = 6 mice/group). Formation of amyloid plaques could not be detected using autoradiography. Compared with sham controls (injection of scramble peptide), significant decrease in [^18^F]FDG uptake was observed in the AD group in the subcortical volume of the ipsilateral hemisphere. Donepezil restored normal glucose metabolism by selectively increasing glucose metabolism in the affected subcortical volume but not in other brain regions. In mice, [^18^F]FDG PET imaging can be optimized to monitor impaired brain function associated with i.c.v injection of Aβ_25–35_, even in the absence of detectable amyloid plaque. This model recapitulates the regional decrease in [^18^F]FDG uptake observed in AD patients. [^18^F]FDG PET imaging can be straightforwardly transferred to AD patients and may aid the development of certain therapies designed to restore the altered brain function in AD.

## Graphical Abstract

[^18^F]2-fluoro-2-deoxy-D-glucose ([^18^F]FDG) PET imaging was performed in a mouse model of Alzheimer’s disease to investigate the impact of donepezil on brain glucose metabolism.

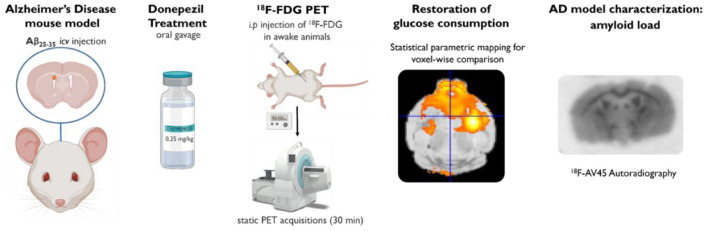

## Introduction

Alzheimer’s disease (AD) is the most prevalent neurodegenerative disease of aging. A large body of research focuses on the development of therapeutic strategies to restore or at least slow down the progression of cognitive decline (Yiannopoulou and Papageorgiou, 2020). Growing understanding of the complex and multifactorial etiology of AD has driven the development of mechanism-based strategies targeting amyloid-beta (Aβ) plaques, Tau protein deposits, apolipoprotein-E (ApoE) function, neuroprotection or neuroinflammation (Cummings et al., 2021). Most animal models of AD have been developed to recapitulate these key histopathological lesions *in vivo* (Götz et al., 2018). These models therefore offer unvaluable tools to test the preclinical efficacy of AD treatments. In this framework, mechanistical proof-of-concept and clinical transfer can be achieved thanks to the availability of translational imaging techniques using corresponding biomarkers (Borsook et al., 2013; Adlard et al., 2014). For instance, Positron Emission Tomography (PET) imaging using radioligands targeting amyloid plaques like [^11^C]labeled Pittsburgh Compound-B ([^11^C]PiB) or fluorinated analogs such as [^18^F]florbetapir has convincingly shown the efficacy of several therapeutic antibodies at decreasing Aβ-load in the brain of animal models of AD and patients (Declercq et al., 2016). However, despite obvious effectiveness at the molecular level, amyloid-targeting therapeutics appear to be ineffective at restoring brain function and cognitive performance in patients with symptomatic AD (Long and Holtzman, 2019; Vaz and Silvestre, 2020).

Sensitivity of the cholinergic system to amyloid toxicity leads to a well characterized impairment of cholinergic neurons within the nucleus basalis magnocellularis, or nucleus of Meynert in human (Emre et al., 1992; Baker-Nigh et al., 2015). The prescription of inhibitors of acetylcholinesterase (AChE) such as donepezil, is therefore considered by clinicians to limit cognitive decline in mild to moderate AD (Knowles, 2006). Specific and reversible inhibition of AChE by donepezil delays acetylcholine hydrolysis and maintains levels of acetylcholine in the synaptic cleft. Donepezil is therefore expected to compensate for the loss of functioning cholinergic neurons observed in AD patients. At the preclinical stage, behavioral tests have clearly shown the positive effect of donepezil on cognitive performance (Meunier et al., 2006; Droguerre et al., 2020). However, in patients, the positive impact of donepezil on cognitive function is difficult to assess (Matsunaga et al., 2019). Translational methods are therefore needed to assess the clinical impact of drugs such as donepezil that aim at restoring normal brain function rather that targeting AD-specific biomarkers, with respect to the progression of the disease in AD patients (Delatour et al., 2010; Cheng et al., 2019).

Learning and memory disorders associated with degeneration of the cholinergic system were confirmed in mice that received intracerebroventricular (i.c.v) injection of the Aβ_25–35_ peptide (Maurice et al., 1996). Using this model, Meunier and colleagues have shown that donepezil is able to alleviate the memory deficits while protecting against Aβ_25–35_ peptide-induced toxicity (Meunier et al., 2006). Although injection of the Aβ_25–35_ peptide has been demonstrated to yield into generation of Aβ fibrils, formation of detectable amyloid plaque may not necessarily appear, thus precluding the use of plaque-targeting PET biomarkers for *in vivo* imaging. Moreover, the link between the intrinsic toxicity of Aβ_25–35_ peptide, the putative formation of senile plaques, and the regional decrease in brain function remains to be investigated as molecular determinants of the positive impact of donepezil.

Brain PET imaging using [^18^F]2-fluoro-2-deoxy-D-glucose ([^18^F]FDG) is now available in most Nuclear Medicine departments to estimate brain glucose metabolism (Varrone et al., 2009). [^18^F]FDG PET is routinely used to support the clinical diagnosis of patients with AD (Jack et al., 2018). In the present study, we hypothesized that [^18^F]FDG PET imaging may provide a translational imaging biomarker of brain function to investigate the impact of chronic donepezil treatment on the regional brain metabolism. [^18^F]FDG PET was therefore evaluated in the awake i.c.v Aβ_25–35_ mouse model and the putative formation of amyloid plaques in this model was investigated using autoradiography.

## Materials and Methods

### Chemicals and Radiochemicals

Donepezil was purchased from Sigma-Aldrich (l’Isle d’Abeau Chesnes, France), [^18^F]2-fluoro 2-deoxy-D-glucose ([^18^F]FDG) for injection was obtained from Cyclopharma (Saint-Beauzire, France). [^18^F]florbetapir ([^18^F]AV45) was produced in-housed (CEA/SHFJ) by the radiochemistry team according to a previously described method (Liu et al., 2010).

### Mouse Model of Alzheimer’s Disease

All procedures were in accordance with European directives on the protection and use of laboratory animals (Council Directive 2010/63/UE, French decree 2013-118). The experimental protocol was evaluated and validated by a local ethic committee for animal use and approved by the French government (*n*° APAFIS#7466-2016110417049220 v3).

Peptide Aβ_25–35_ and scramble peptide (Sc.) were purchased from Genepep (Saint-Jean-de-Védas, France). Eighteen 6-week-old Swiss male mice (body weight: 30–35 g) were anesthetized with isoflurane 2.5% and received unilateral intracerebroventricular (i.c.v) injection of 9 nmol/3 μL per mouse of Aβ_25–35_ peptide or Sc in the right hemisphere, as previously described (Maurice et al., 1996). Animals were maintained under temperature-controlled (range 20–24°C) conditions and a 12:12 h light-dark cycle. Mice were housed by groups of 5 in Plexiglas cages with *ad libitum* access to water and standard diet and enrichment.

### Autoradiographic Characterization of the Alzheimer’s Disease Model

Density of amyloid plaques in the AD model was estimated using *in vitro* autoradiography of mouse brains resected 10 days after i.c.v injection of Aβ_25–35_, using the amyloid radioligand [^18^F]florbetapir (Choi et al., 2009). Brain slices (20 μm) were obtained using a cryostat (Leica CM3050 S, Nanterre, France). Slices were incubated for 30 min with [^18^F]florbetapir (1,158 mCi/200 mL; Molar activity: 2,112 mCi/μmol) in *Tris* Buffer [50 mM TRIZMA, from Sigma-Aldrich (l’Isle d’Abeau Chesnes, France)] adjusted to pH 7.4 with NaCl 0.9% at pH = 7.4. Unbound excess ligand was removed by two subsequent 2 min wash cycles in cold buffer followed by a final rinse in cold deionized water. Then, brain sections were placed in direct contact with a Phospho-Imager screen (Molecular Dynamics, Sunnyvale, CA, United States) and exposed for 4 h. Autoradiograms in control and AD group were analyzed using ImageJ software.^1^ [^18^F]florbetapir binding was quantified by delineating regions of interest (ROIs) on four brain sections per animal in four animals of the control and the AD group. Mean gray value for each ROI normalized by area was obtained and statistically compared. The autoradiography method was validated in TgF344 rat, used as a positive control for brain accumulation of amyloid deposit (Cohen et al., 2013). Validation data are reported as Supplementary Figure 1.

### Positron Emission Tomography Study Groups

Three groups of mice (*n* = 6) were defined as follows: (i) Sc.-injected mice (*control*); (ii) Aβ_25–35_ peptide injected mice treated with vehicle (*AD*); (iii) Aβ_25–35_ peptide injected mice treated with donepezil (0.25 mg/kg) (*AD* + *DPZ*). Treatment started 3 days after i.c.v injection of Aβ_25–35_ peptide, for 7 consecutive days. Vehicle solution consisted in dimethyl sulfoxide (DMSO) 2% in water. AD mice were treated by donepezil or vehicle once daily by oral gavage (5 mL/kg).

### [^18^F]2-Fluoro-2-Deoxy-D-Glucose Positron Emission Tomography Imaging Acquisition

[^18^F]2-fluoro-2-deoxy-D-glucose PET was performed after 7 days treatment, i.e., 10 days after i.c.v injection of Aβ_25–35_ peptide. The last dose of treatment was administered in the morning, 7 h before PET. Then, mice were fasted until [^18^F]FDG PET acquisition, and only water was given to animals in the meantime.

A customized PET protocol was used to limit the impact of anesthesia on the estimation of brain function using [^18^F]FDG PET (Toyama et al., 2004). Briefly, [^18^F]FDG (0.2 mL; mean dose = 7.2 ± 0.5 MBq) was injected intraperitoneally (IP) in awake mice (Schiffer et al., 2007). Mice were then transferred in a cage for 40 min to allow for the brain uptake of [^18^F]FDG in awake and freely moving animals. Then, anesthesia was induced using isoflurane 2–2.5% in O_2_. Anesthetized animals were rapidly transferred under the microPET scanner the brain in the middle of the field of view (Inveon, microPET; spatial resolution 1.6 mm; Siemens Healthcare, Knoxville, TN, United States). A static 30 min PET acquisition was performed 50 min after [^18^F]FDG injection under anesthesia maintained by a facial mask (1.5–2% isoflurane). Static brain PET acquisition was performed at the plateau of [^18^F]FDG brain kinetics after IP injection, assuming limited impact of isoflurane anesthesia on the time-course of [^18^F]FDG uptake by the brain (Toyama et al., 2004; Fueger et al., 2006).

### [^18^F]2-Fluoro-2-Deoxy-D-Glucose Positron Emission Tomography Data Analysis

Static PET images were reconstructed by the 2D OSEM/FORE algorithm and corrected for attenuation, random coincidences and scatter. The voxel size was 0.2 mm × 0.2 mm × 0.2 mm. Brain PET images were corrected for radioactive decay, injected dose and body weight and was expressed as standardized uptake values (SUV), with SUV = tissue activity (kBq/cc)/[injected dose (kBq)/body weight (g)]. SUV-normalized PET images were spatially co-registered to a standard mouse [^18^F]FDG PET template (Schiffer et al., 2007) using Pmod software (version 3.8, PMOD Technologies Ltd., Zurich, Switzerland). Quantitative SUV values were determined through a volume-of-interest (VOI) analysis in selected brain regions which size is relevant to the spatial resolution of the microPET scanner (∼1.4 mm), which included thalamus, cerebellum, brain stem, striatum, hippocampus, hypothalamus, amygdala, midbrain, cortex and whole brain. Regional PET data and PET SUV-normalized PET images were then divided by each individual SUV values obtained in the cerebellum. The cerebellum was selected as a reference region due to its low involvement in AD pathology (Talbot et al., 1994), to obtain PET data and images with lower variability than SUV (Goutal et al., 2020). Then, a brain mask was applied on normalized PET images to keep cerebral voxels only.

Mean regional SUV and SUVR values obtained in each group were statistically compared.

Then, parametric SUVR images were compared using Statistical Parametric Mapping software (SPM8), a one-way ANOVA test was applied with a significance threshold of *p* < 0.05 and with an extend threshold of 200 voxels, uncorrected for multiple comparison (Vodovar et al., 2018). Then, statistical parametric maps were overlaid on mouse MRI template (Schiffer et al., 2007) to visualize which brain regions are affected by statistical differences on [^18^F]FDG uptake.

### Statistical Analysis

A student’s *t*-test was performed to compare [^18^F]florbetapir autoradiography data in control and AD animals. A one-way ANOVA was performed to compare SUV and SUVR values between groups for each brain regions. Statistical analysis was performed using GraphPad software (version 9.0).

## Results

### [^18^F]florbetapir Autoradiography

**FIGURE 1:**
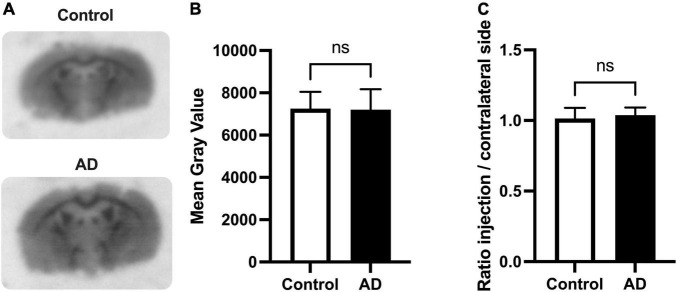
Autoradiography of brain slices using the amyloid radioligand [^18^F]florbetapir in the mouse model of Alzheimer’s disease (AD). Brains were resected 10 days after intracerebroventricular injection (i.c.v) of amyloid peptide Aβ_25–35_. Representative mouse brain sections of [^18^F]florbetapir binding within hippocampal and cortical region is shown in control and AD mice (in **A**). Quantitative *in vitro* analysis of [^18^F]florbetapir binding on autoradiograms of the i.c.v Aβ_25–35_ mouse model was performed using *n* = 4 animals per group with four quantified brain sections each. Mean gray values normalized by regions of interest (ROI) area are presented **(B)**. Ratios of [^18^F]florbetapir binding on injection side (ipsilateral, right) to contralateral side (left) is shown in panel **(C)**. No significant differences in [^18^F]florbetapir binding *in vitro* was measured between control and AD group (ns; *p* > 0.05).

Putative amyloid load associated with the i.c.v injection of Aβ_25–35_ peptide was first investigated using autoradiography with the amyloid radioligand [^18^F]florbetapir. In control (Sc-injected) mice; higher binding was observed in white matter regions (e.g., corpus callosum) compared to gray matter regions, consistent with the brain binding of [^18^F]florbetapir to myelin (Auvity et al., 2020; Figure 1A). No differences in [^18^F]florbetapir binding could be visually observed between brain slices obtained from animals of the AD group compared with corresponding slices of the control group. Neither was visual differences between the ipsi- and contralateral brain hemispheres of AD mice. Quantification confirmed these observations and no significant difference could be found in the radioactivity measured in the ipsilateral hemisphere of AD mice compared with the contralateral hemisphere, or compared with the brain of control mice (*p* > 0.05) (Figures 1B,C).

We first hypothesized that the absence of detectable amyloid plaques in the mouse model of i.c.v injection of Aβ_25–35_ peptide may be due to insufficient sensitivity of the autoradiographic method. Sensitivity of our [^18^F]florbetapir autoradiographic condition was tested on brain slices from TgF344 rats, known to form amyloid plaques and serving as positive control. Autoradiography data confirmed the presence of amyloid plaques after their incubation with [^18^F]florbetapir and high radiotracer binding was detected in cortical and hippocampus regions in AD rats but not in wild-type rats (Supplementary Figure 1).

### [^18^F]2-Fluoro-2-Deoxy-D-Glucose Brain Positron Emission Tomography Imaging

**FIGURE 2:**
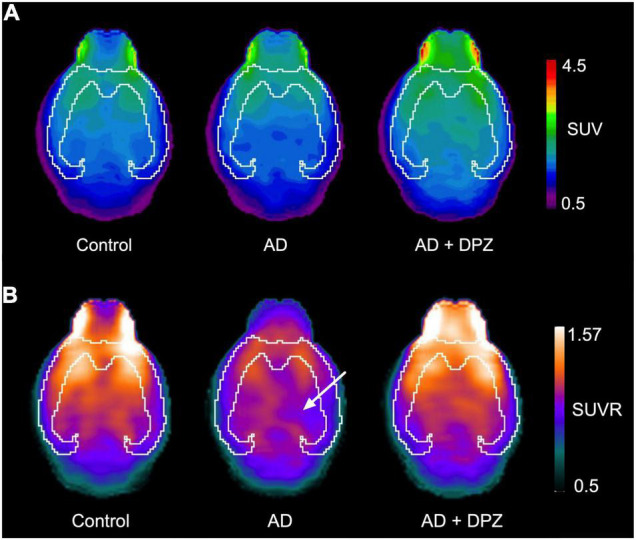
Representative brain [^18^F]2-fluoro-2-deoxy-D-glucose ([^18^F]FDG) positron emission tomography (PET) images obtained in mice. In panel **(A)**, [^18^F]FDG PET image is expressed as standardized uptake values (SUV), with SUV = tissue activity (kBq/cc)/[injected dose (kBq)/body weight (g)]. Uptake was determined in control mice, in a mouse model of Alzheimer’s disease (AD) consisting in intracerebroventricular (i.c.v) injection of amyloid peptide Aβ_25–35_ (AD) and in mice that received i.c.v injection of amyloid peptide Aβ_25–35_ treated with donepezil (AD + DPZ). Corresponding parametric SUVR images (PET signal is normalized by cerebellar uptake of radioactivity) are shown in panel **(B)**. The cortical volume in delineated in white. A white arrow shows the subcortical volume with visually decreased SUVR in the right ipsilateral hemisphere compared with the contralateral hemisphere.

**FIGURE 3:**
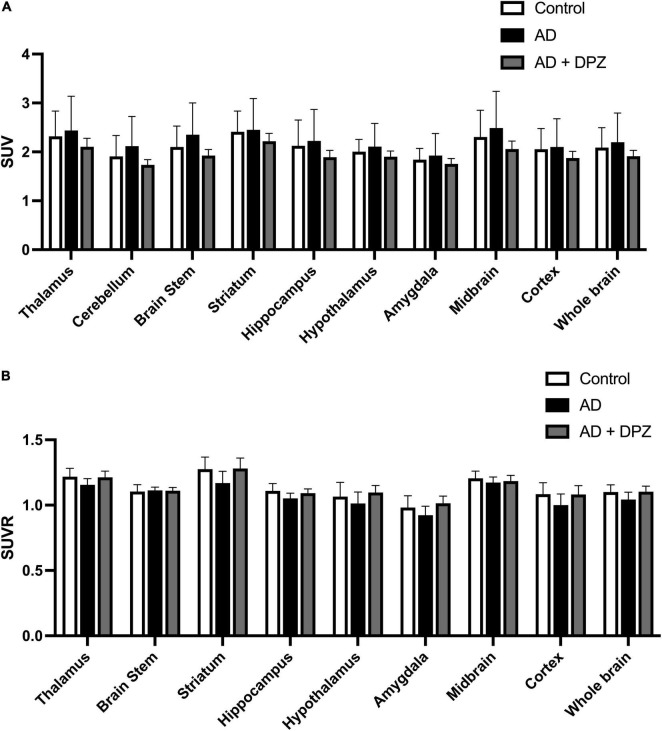
Quantitative brain [^18^F]2-fluoro-2-deoxy-D-glucose ([^18^F]FDG) positron emission tomography (PET) data in selected brain region in mice. [^18^F]FDG brain uptake was expressed as standardized uptake values (SUV). Uptake was determined in control mice, in a mouse model of Alzheimer’s disease (AD) consisting in intracerebroventricular (i.c.v) injection of amyloid peptide Aβ_25–35_ (AD) and in mice that received i.c.v injection of amyloid peptide Aβ_25–35_ treated with donepezil (AD + DPZ). Data are reported as mean ± S.D (*n* = 6 per group). There were statistical differences on neither SUV values (in **A**) nor SUV values normalized by cerebellum uptake (SUVR, in **B**) between groups (ns; *p* > 0.05).

Cerebral glucose metabolism was investigated using [^18^F]FDG PET. Visual inspection of brain SUV PET images did not clearly show a decrease in brain uptake (Figure 2A). Consistently, no significant differences in [^18^F]FDG uptake have been found between groups of mice for the predefined VOIs (*p* > 0.1, Figure 3A). The coefficient of variation (CV = S.D/mean) of SUV values in the whole-brain was 19.5% for control mice, 27.2% for AD mice and 6.2% for AD + DPZ mice. Parametric SUVR images normalized to the cerebellum as a reference region were generated to reduce the variability (Rojas et al., 2013; Brendel et al., 2016). A cluster of decreased [^18^F]FDG could be visually observed in a subcortical volume of the ipsilateral hemisphere compared with the contralateral volume (Figure 2B). The CV of SUVR values in the whole-brain was 5.1% for control mice, 5.4% for AD mice and 3.9% for AD + DPZ mice. However, no significant differences could be detected when comparing mean SUVR values in the predefined brain VOI of the template (*p* > 0.1, Figure 3B).

**FIGURE 4:**
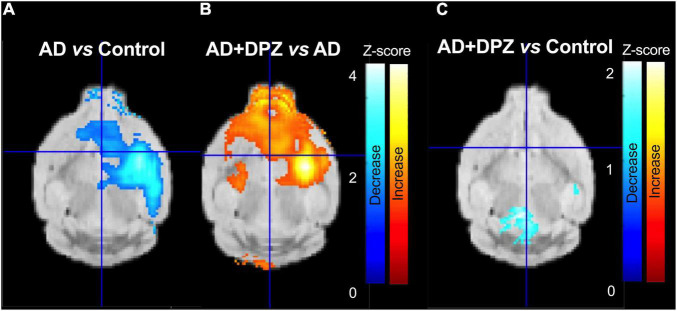
Parametric mapping of [^18^F]2-fluoro-2-deoxy-D-glucose ([^18^F]FDG) brain uptake reflecting therapeutic response to donepezil. Positron emission tomography (PET) images in Standard Uptake Value (SUV) were normalized to radioactivity in the cerebellum. Statistical maps were overlaid to a mouse MRI template. Panel **(A)** shows regions with significant decrease in glucose metabolism (*p* < 0.05; threshold: 200 voxels) in a mouse model of Alzheimer’s disease (AD) obtained after unilateral intracerebroventricular injection of peptide Aβ_25–35_ compared with control mice. Region with significant increase in brain glucose metabolism after DPZ (0.25 mg/kg daily, 7 days) treatment mice in shown in panel **(B)** (*p* < 0.05; threshold: 200 voxels). No differences was observed in the right hemisphere between control and AD mice treated with DPZ [0.25 mg/kg, in panel **(C)**].

We therefore performed a statistical parametric mapping (SPM) method to allow for a voxel-wise comparison (threshold *p* < 0.05, extent threshold 200 voxels) with no regional *a priori*. Using this SPM approach on SUVR images, a cluster of significantly decreased [^18^F]FDG uptake was observed in the AD group as compared to control mice. This cluster, located to the right mouse brain hemisphere, in the subcortical area, was consistent with the unilateral i.c.v injection of Aβ_25–35_ peptide (Figure 4A).

The same brain volume was associated with most significant increase in [^18^F]FDG uptake in AD mice treated with donepezil as compared to vehicle-treated AD mice. Consistently, [^18^F]FDG uptake in AD + DPZ mice was not significantly different compared to the control group, suggesting local loss in cerebral glucose metabolism was restored by donepezil (Figure 4).

## Discussion

In the present study, regional decline in brain function associated with i.c.v injection of Aβ_25–35_ peptide was assessed using minimally invasive [^18^F]FDG PET imaging. Local decrease in brain glucose metabolism was significant in the area surrounding the unilateral injection of Aβ_25–35_ and was not associated with the presence of detectable amyloid plaques. Interestingly, normal glucose metabolism in this region was restored by therapeutic dose of the AChE inhibitor donepezil.

[^18^F]2-fluoro-2-deoxy-D-glucose PET is routinely performed in hospital setting as a biomarker of neurodegeneration in AD (Silverman et al., 1999). [^18^F]FDG PET is part of the classification according to the stages of AD progression. In MCI and AD patients, an early decrease in [^18^F]FDG uptake is observed in temporal brain areas associated with memory processes (hippocampus) (Mosconi, 2005; Mosconi et al., 2005). In more advanced stages of the disease, lower glucose metabolism reaches the cortical areas, particularly the frontal cortex. Some brain regions are spared by the reduction in glucose consumption, such as the visual and primary motor cortices as well as the cerebellum. Despite clinical observations suggesting a decrease in glucose consumption in the brain of AD patients, preclinical studies using mouse models of Alzheimer’s disease remain contradictory. Indeed, glucose consumption was higher, unchanged or lower with respect to the different mouse models and quantification methods (Bouter and Bouter, 2019). Moreover, all preclinical studies using the cerebellum as a reference region reported an increase in glucose consumption (Poisnel et al., 2012; Waldron et al., 2015; Brendel et al., 2016), which is not consistent with clinical observations in any stage of AD. Interestingly in this i.c.v mouse model of AD, the use of cerebellum to normalize PET images unveiled a decrease in brain glucose metabolism, which better recapitulates clinical observation.

It remains unclear whether impaired glucose metabolism within the CNS precedes the onset of amyloid pathology (formation of amyloid plaques) and the development of neurodegenerative processes in AD (Foster et al., 2008). No correlation was found between the decrease of [^18^F]FDG binding and amyloid load within brain regions in a transgenic mouse model (Waldron et al., 2015). [^18^F]florbetapir *ex vivo* binding was not correlated with glucose consumption (Waldron et al., 2017). These observations are consistent with our [^18^F]FDG PET data showing decline in glucose metabolism associated with Aβ_25–35_ peptide injection in mice, with no detectable increase in amyloid load.

In the i.c.v model of AD, detection of fibrillar forms of the Aβ_25–35_ peptide similar to the amyloid deposits of senile plaques was demonstrated using congo red staining (Maurice et al., 1996). In rats with cerebral injection of Aβ_25–35_, experiments using electron microscopy, infrared spectroscopy and congo red staining validated the aggregation of Aβ_25–35_ peptide into amyloid fibrils (Zussy et al., 2011). This suggests that direct neuronal toxicity of Aβ_25–35_ oligomers, with detectable impact on brain function, may precede the formation of detectable amyloid plaques. Our *ex vivo* autoradiography may, however, lack the sensitivity to detect low levels of amyloid plaques. Further experiments using optimized autoradiography conditions, other radioligands or more sensitive methods such as synchrotron-based X-ray phase contrast (Astolfo et al., 2016) may be necessary to conclude to the presence of the absence of amyloid plaques 10 days after i.c.v injection of Aβ_25–35_ peptide in this model.

Beyond diagnostic use, [^18^F]FDG PET provides a convenient functional neuroimaging biomarker of neuronal activity (Varrone et al., 2009). We hypothesized that the effectiveness of symptomatic therapies in AD could be assessed using [^18^F]FDG PET (Vodovar et al., 2018).

An optimized acquisition procedure was performed to limit the impact of anesthesia on brain function and metabolism. We took advantage of the poorly reversible uptake of [^18^F]FDG in the brain cells, which leads to a plateau in the brain kinetics of radioactivity. Previous studies have shown that the brain uptake of [^18^F]FDG is not impacted by anesthesia when PET acquisition starts 40 min after intravenous [^18^F]FDG injection, reaching similar levels than brain uptake measured in conscious (non-anesthetized) rats (Matsumura et al., 2003). [^18^F]FDG was injected intraperitoneally to limit the impact of stress induced by the handling of awake animals on brain function (Fueger et al., 2006). The time-course of [^18^F]FDG brain kinetics after IP administration was assessed in anesthetized mice to define the optimal acquisition time frame as has been investigated previously by others (Toyama et al., 2004; Fueger et al., 2006). The AD model using i.c.v injection of Aβ_25–35_ presents well characterized memory disorders (Maurice et al., 1996; D’Agostino et al., 2012). Our results suggests that [^18^F]FDG brain uptake may quantitatively monitor functional loss in neuronal decline activity. Other pharmacological imaging techniques such as functional MRI (pharmacological MRI) and functional ultrasound (pharmaco-FUS) are being developed to investigate the CNS impact of mechanistic and non-mechanistic therapies in AD (Tournier et al., 2020; Vidal et al., 2020). This work illustrates the added value of [^18^F]FDG PET which can be performed as a minimally invasive and translational alternative to limit the impact of anesthesia on the estimation of the effects of investigated drugs in brain regions.

Positive effect of chronic donepezil on cognitive performance in mice models of AD has been reported from 1 h (acute, single dose) (Nagakura et al., 2013), 4 days (daily dose) (Shin et al., 2018) to 14 days (daily dose) after initiation of the treatment (Droguerre et al., 2020). In the present study chronic administration of donepezil (0.25 mg/kg daily) for 7 days restored normal brain metabolism in a mouse model of AD. The results are consistent with the increase in [^18^F]FDG brain uptake reported after donepezil treatment (3 mg/day for the first 2 weeks followed by 5 mg/day if tolerated) in AD patients (Shimada et al., 2011). Interestingly, increased brain metabolism induced by donepezil in AD mice was targeted to the region with decreased glucose metabolism compared with control mice. This suggests that donepezil preferentially acts at restoring impaired glucose metabolism induced by Aβ_25–35_-mediated toxicity rather than enhancing global brain function. Indeed, no increase in brain metabolism was observed in the whole brain of donepezil-treated AD mice compared with controls (Figures 2–4). It may be hypothesized that effects of donepezil other than its action on AChE may occur. It was recently reported that donepezil may alleviate Aβ-induced microglial and astrocytic activation, and reduce disease induced neuroinflammation in a transgenic mouse model of AD (Kim et al., 2021).

Beyond its use in the treatment of AD, a growing body of research suggest that donepezil might also be beneficial in the treatment of orphan disease such as Dravet syndrome or Niemann-Pick disease type C (Seo et al., 2014; Wong et al., 2019). Further experiments are, however, needed to test the relevance of [^18^F]FDG PET as an translational imaging biomarker to assess and optimize the beneficial effects of donepezil in animal models of these diseases and patients.

The present studies investigated brain [^18^F]FDG PET as a translational imaging biomarker in the AD model of i.c.v injection of Aβ_25–35_ in mice. This model benefits from extensive behavioral characterization with well described cognitive and memory loss. Moreover, our results suggests that regional the decrease in [^18^F]FDG uptake in mice may be used as a model for the decreased brain glucose metabolism observed in patients. However, the decrease in brain glucose metabolism, consistent with cognitive decline, was observed in mice in the absence or before the formation of detectable amyloid plaque. This may not accurately recapitulate the complexity of the disease observed in humans, which includes formation of amyloid plaques, Tau neurofibrillary tangles and other well-established biomarkers of the disease (Jack et al., 2018). Our results suggest that brain [^18^F]FDG PET in this mouse model, associated with other target-specific biomarkers, may aid the preclinical evaluation of AD therapies to restore normal neuronal function.

## Data Availability Statement

The raw data supporting the conclusions of this article will be made available by the authors, without undue reservation.

## Ethics Statement

The animal study was reviewed and approved by the CETEA-044-CEA DSV IdF.

## Author Contributions

NT, MD, MC, and AW designed the experiments. GH, SG, FC, AW, and NT performed the experiments. GH, SG, and AW analyzed the data. MS, MB, and MC contributed to the interpretation of the results. GH wrote the first draft of the manuscript. All authors contributed to manuscript revision, read, and approved the submitted version.

## Conflict of Interest

MD and MC were full-time employees of Theranexus Company. The authors declare that this study received funding from Theranexus Company. The funder had the following involvement in the study: Study design, interpretation of the results. The remaining authors declare that the research was conducted in the absence of any commercial or financial relationships that could be construed as a potential conflict of interest.

## Publisher’s Note

All claims expressed in this article are solely those of the authors and do not necessarily represent those of their affiliated organizations, or those of the publisher, the editors and the reviewers. Any product that may be evaluated in this article, or claim that may be made by its manufacturer, is not guaranteed or endorsed by the publisher.

## Footnotes

1
https://imagej.nih.gov
