# Supplementary material for: Impact of Donepezil on Brain Glucose Metabolism Assessed Using [18F]2-Fluoro-2-deoxy-D-Glucose Positron Emission Tomography Imaging in a Mouse Model of Alzheimer’s Disease Induced by Intracerebroventricular Injection of Amyloid-Beta Peptide
Source: Front Neurosci. 2022 Feb 25;16:835577. doi: 10.3389/fnins.2022.835577 (PMC8916213; doi:10.3389/fnins.2022.835577)

Supplementary Material

Supplementary Figure

Supplementary Figure S1. Representative TgF344-AD brain sections of [^{18}F]forbetapir binding in wild-type rats and the TgF344 model of Alzheimer disease. The TgF344-AD model (F344-Tg(Prp-APP,Prp-PS1)19/Rrrc) is known to produce amyloid Aβ plaques. This model was used as a positive control to validate [^{18}F]forbetapir autoradiography. Binding of [^{18}F]forbetapir was spread in hippocampal and cortical region in this model consistent with the accumulation of Aβ plaques in this model. In corresponding wild-type rats of the same age, much lower radiotracer uptake was observed in these regions.

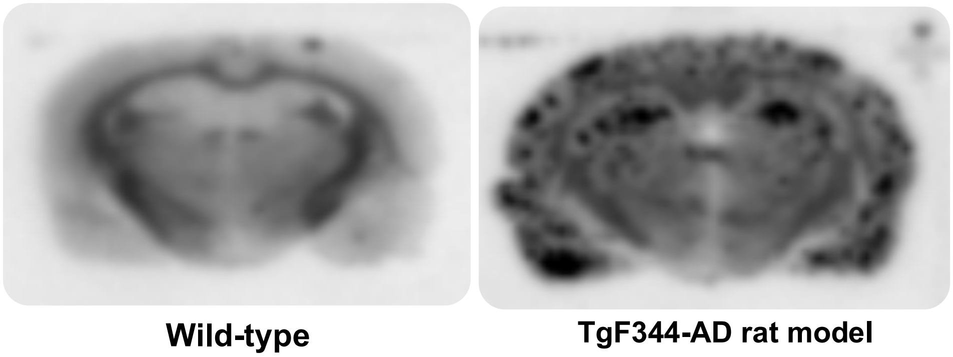

Supplement: Supplementary file 1 [file Data_Sheet_1.PDF]
